# *In Situ* Formation of Bi_2_MoO_6_-Bi_2_S_3_ Heterostructure: A Proof-Of-Concept Study for Photoelectrochemical Bioassay of l-Cysteine

**DOI:** 10.3389/fchem.2022.845617

**Published:** 2022-05-18

**Authors:** Hui-Jin Xiao, Xiao-Jing Liao, Hui Wang, Shu-Wei Ren, Jun-Tao Cao, Yan-Ming Liu

**Affiliations:** ^1^ Xinyang Key Laboratory of Functional Nanomaterials for Bioanalysis, College of Chemistry and Chemical Engineering, Xinyang Normal University, Xinyang, China; ^2^ Xinyang Central Hospital, Xinyang, China

**Keywords:** photoelectrochemical sensor, Bi_2_MoO_6_–Bi_2_S_3_ heterostructure, L-cysteine, *in situ* formation reaction, ion exchange reaction

## Abstract

A novel signal-increased photoelectrochemical (PEC) biosensor for l-cysteine (L-Cys) was proposed based on the Bi_2_MoO_6_–Bi_2_S_3_ heterostructure formed *in situ* on the indium–tin oxide (ITO) electrode. To fabricate the PEC biosensor, Bi_2_MoO_6_ nanoparticles were prepared by a hydrothermal method and coated on a bare ITO electrode. When L-Cys existed, Bi_2_S_3_ was formed *in situ* on the interface of the Bi_2_MoO_6_/ITO electrode by a chemical displacement reaction. Under the visible light irradiation, the Bi_2_MoO_6_–Bi_2_S_3_/ITO electrode exhibited evident enhancement in photocurrent response compared with the Bi_2_MoO_6_/ITO electrode, owing to the signal-increased sensing system and the excellent property of the formed Bi_2_MoO_6_–Bi_2_S_3_ heterostructure such as the widened light absorption range and efficient separation of photo-induced electron–hole pairs. Under the optimal conditions, the sensor for L-Cys detection has a linear range from 5.0 × 10^−11^ to 1.0 × 10^−4^ mol L^−1^ and a detection limit of 5.0 × 10^−12^ mol L^−1^. The recoveries ranging from 90.0% to 110.0% for determining L-Cys in human serum samples validated the applicability of the biosensor. This strategy not only provides a method for L-Cys detection but also broadens the application of the PEC bioanalysis based on *in situ* formation of photoactive materials.

## Introduction

l-Cysteine (L-Cys), which is involved in the process of protein synthesis, affects the function of protein and plays an important role in the life system (Palego et al., 2015). Its abnormal levels in human serum are associated with lots of diseases, and thus it is considered a significant biomarker. For instance, people with heart disease and liver injury often have low levels of L-Cys in their blood (Wu et al., 2016), whereas people with Alzheimer’s disease and cancer often have high levels of L-Cys (Li et al., 2014b; Huang et al., 2018). Therefore, monitoring the content of L-Cys in human body is meaningful. Currently, some analytical methods such as high-performance liquid chromatography (Deáková et al., 2015), mass spectrometry (Li et al., 2014a), fluorescence (Li et al., 2019), colorimetry (Song et al., 2018), and photoelectrochemistry (PEC) (Peng et al., 2020) have been developed for L-Cys detection.

PEC analysis, a fast, efficient, and low background analytical method, has attracted great attention in recent years (Cao et al., 2021; Lv et al., 2021; Zhu et al., 2021). Until now, many sensing principles have been exploited and adopted for the PEC bioanalysis, such as steric hindrance effect (Wang et al., 2019c; Meng et al., 2020), electron donor/acceptor reaction (Li et al., 2017; Wang et al., 2019b), exciton–plasmon interactions (Ma et al., 2016; Dong et al., 2017), plasmon-enhanced effect (Li et al., 2016; Qiu et al., 2018), and *in situ* growth reaction (Qiu and Tang, 2020). Of these, the signaling mechanism based on the *in situ* growth reaction that acts directly on the electrode is not only simple to operate but also with a low background signal (Hou et al., 2016). For example, on the basis of the reaction between L-Cys and copper compounds, Zhu et al. (2017) constructed a PEC bioassay of L-Cys using a CuO–Cu_2_O heterojunction as a photoactive material. By using the reaction between Cu^2+^ and S^2−^ from the WO_3_–Au–CdS nanocomposite, Zhang et al. (2019) designed a PEC immunoassay for the prostate-specific antigen. However, these works have always quantified the targets based on the signal decrease, which limits the sensitivity to some extent. By the reaction between Ag^+^ and BiOI/Ni electrode, Yu et al. (2019a) constructed a signal-increased biosensing system. In this system, the AgI–Ag–BiOI Z-scheme heterojunction formed *in situ* greatly enhanced the PEC response, achieving satisfied detection sensitivity and stability. Considering the good performance and the few reports of such strategy, exploiting the new *in situ* growth reaction to construct signal-increased sensing systems and extending their applications in PEC bioanalysis are urgent and necessary.

Among various semiconductor materials, bismuth-based semiconductors possess advantages of good biocompatibility and highly visible light response (Chen et al., 2016; Zhou et al., 2017; Yu et al., 2019b). Bi_2_MoO_6_, featuring non-toxic, good stability, and adjustable morphology (Li et al., 2020), has attracted wide attention. In addition, Bi_2_MoO_6_ has a layered structure with a [Bi_2_O_2_]^2+^ layer stuck between two MoO_4_^2−^ slabs, which makes it have lots of active surfaces (Wu et al., 2018), while the PEC performance of Bi_2_MoO_6_ leaves much to be desired due to the rapid recombination between holes and electrons. In order to restrain such recombination, constructing heterostructures is one of the most effective strategies (Wang et al., 2019a; Liao et al., 2021). As a method to form heterojunctions, ion exchange can be excited by the differences in solubility of different substances and helps maintain their original state to a large extent (Wang et al., 2017). Intelligently, both Bi_2_MoO_6_ and Bi_2_S_3_ contain bismuth element, and the solubility of Bi_2_S_3_ is far less than that of Bi_2_MoO_6_. Based on this, whether the principle of the ion exchange reaction can be used for *in situ* generation of Bi_2_MoO_6_–Bi_2_S_3_ heterostructure and construction of a PEC biosensor?

**SCHEME 1:**
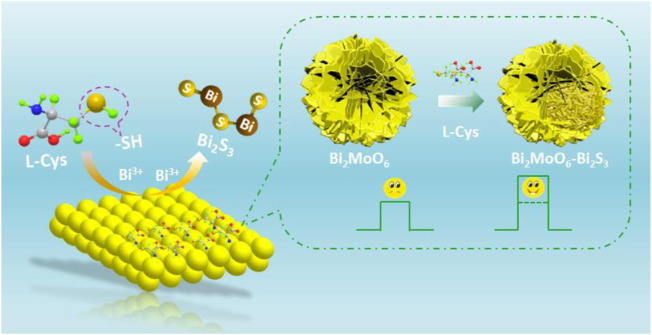
Illustration of the proposed PEC sensor.

A signal-increased PEC biosensor for L-Cys detection was proposed based on the *in situ* formation of a Bi_2_MoO_6_–Bi_2_S_3_ heterostructure on the indium–tin oxide (ITO) electrode. As illustrated in Scheme 1, Bi_2_MoO_6_ nanoparticles were initially coated on a bare ITO electrode. In the existence of L-Cys, Bi_2_S_3_ was generated *in situ* on the interface of Bi_2_MoO_6_/ITO by a chemical displacement reaction between sulfur ions from L-Cys and MoO_6_^6−^ from Bi_2_MoO_6_. The compact contact and the matchable band-edge levels of Bi_2_MoO_6_ and Bi_2_S_3_ formed a heterostructure, which broadens the light absorption range and effectively restrains the electron–hole recombination, producing an improved photocurrent response. The increased concentrations of L-Cys could generate more amount of Bi_2_S_3_ on the Bi_2_MoO_6_/ITO interface, thereby boosting the photocurrent response. By this means, a signal-increased PEC system to quantitatively detect L-Cys was established by measuring the photocurrent change of the photoelectrode.

## Experimental

### Chemicals and Reagents

Bismuth nitrate (Bi(NO_3_)_3_·5H_2_O), ethylene glycol (EG), and sodium molybdate (Na_2_MoO_4_·2H_2_O) were purchased from Macklin Biochemical Co., Ltd. (Shanghai, China). l-Serine (L-Ser), glycine (Gly), and l-tyrosine (L-Tyr) were purchased from Sinopharm Chemical Reagent Co., Ltd. (China). L-Cys and glutathione (GSH) were obtained from Aladdin Reagent Inc. (Shanghai, China). Ascorbic acid (AA), sodium sulfate (Na_2_SO_4_), and sodium sulfite (Na_2_SO_3_) were purchased from Sinopharm Chemical Reagent Co., Ltd. (China). Phosphate buffer solution of 0.01 M (PBS, pH 7.4) was prepared with NaH_2_PO_4_.2H_2_O, K_2_HPO_4_.3H_2_O, and KCl. All chemical reagents were of analytical grade, and all aqueous solutions were prepared with ultrapure water (18.2 MΩ cm).

### Apparatus

The PEC system consists of a CHI660E electrochemical workstation (Shanghai Chenhua Apparatus Corporation, China) and a PEAC 200A PEC reaction instrument (Tianjin Aidahengsheng Science-Technology Development Co., Ltd., China). PEC experiments and linear sweep voltammetry (LSV) curves were conducted on the PEC system using a three-electrode system: an ITO electrode with a geometric area of 0.25 cm^2^ as the working electrode, a saturated Ag/AgCl electrode as the reference electrode, and a Pt wire as the counter electrode. The electrochemical impedance spectra (EIS) were implemented on a CHI660E electrochemical workstation in 5.0 mM K_3_ [Fe(CN)_6_]**/**K_4_ [Fe(CN)_6_] solution containing 0.1 M KCl. The scanning electron microscope (SEM) images were acquired from the Hitachi S-4800 SEM (Tokyo, Japan). UV-visible diffuse reflection spectra were recorded using a PerkinElmer Lambda 950 UV-visible spectrophotometer (United States). X-ray photoelectron spectroscopy (XPS) images were recorded on a K-Alpha X-ray photoelectron spectrometer (Thermo Fisher Scientific Co., Waltham, MA, United States). Fourier transform infrared (FT-IR) spectra were acquired from the Bruker TENZOR 27 spectrophotometer (Bruker Optics, Germany).

### Synthesis of Bi_2_MoO_6_ Nanoparticles

Bi_2_MoO_6_ was synthesized by a hydrothermal method (Dai et al., 2018). First, 0.4210 g of Na_2_MoO_4_·2H_2_O was dissolved in 5 ml of EG under stirring for 0.5 h, and 1.6866 g of Bi(NO_3_)_3_·5H_2_O solution was prepared in the same way. After mixing them together, 20 ml of ethanol was added dropwise under stirring. Second, the resulted solution was transferred into the Teflon-lined stainless steel autoclave, heated to 160°C for 12 h, and cooled to room temperature. Finally, the resultant product collected by centrifugation was washed three times with ethanol as well as water, dried overnight at 80°C, and then annealed at 400°C for 3 h to obtain Bi_2_MoO_6_ nanoparticles.

### Fabrication of the Photoelectrochemical Biosensor

Bi_2_MoO_6_ suspension of 20 microliters with a concentration of 3 mg ml^−1^ was evenly dropped onto the cleaned ITO electrode and dried at 60°C for 20 min. Afterward, 20 µL of L-Cys solution was cast onto the surface of Bi_2_MoO_6_/ITO gently. After the reaction at 37°C for 0.5 h, the electrode was washed with water and then immersed in 0.01 M PBS (pH 7.4) containing 0.1 M AA for PEC measurement.

## Results and Discussion

### Material Characterization

**FIGURE 1:**
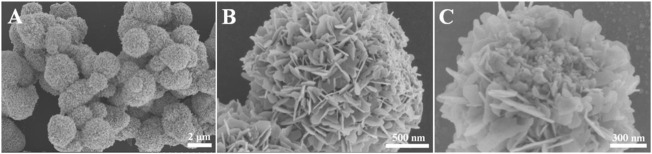
SEM images of Bi_2_MoO_6_ **(A,B)** and Bi_2_MoO_6_ after reacting with L-Cys **(C)**.

The morphology of Bi_2_MoO_6_ was characterized using the SEM. Figures 1A,B depicted that Bi_2_MoO_6_ possessed a nanosheet-assembled spherical structure, and the diameters of the microsphere were less than 3 µm. The stacked sheet structure makes the material have a large specific surface area, which benefits for the subsequent ion exchange reaction and the PEC detection. After incubated with L-Cys, parts of nanosheets granulated on the microsphere of Bi_2_MoO_6_ (Figure 1C), indicating the interaction between Bi_2_MoO_6_ and L-Cys. Additionally, the elemental mapping images in Supplementary Figure S1 suggested that Bi, Mo, O, and S elements existed in the material, indicating the reaction between Bi_2_MoO_6_ and L-Cys.

**FIGURE 2:**
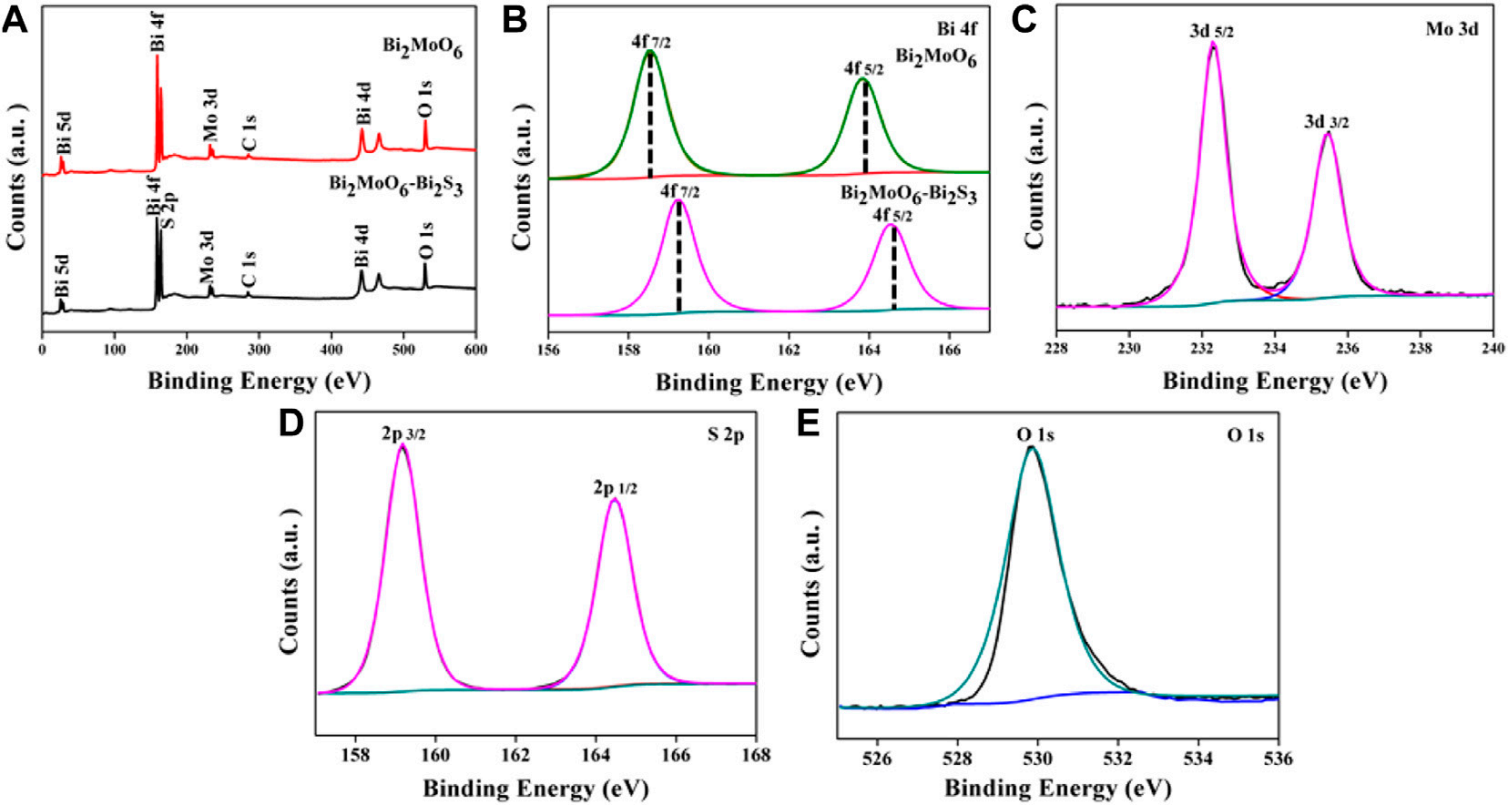
XPS survey spectra of Bi_2_MoO_6_ before and after reacting with L-Cys **(A)**; high-resolution XPS spectra of Bi 4f **(B)**, Mo 3d **(C)**, S 2p **(D)**, and O 1s **(E)**.

To characterize the chemical composition and chemical state of Bi_2_MoO_6_ before and after reacting with L-Cys, XPS analysis was performed. As shown in Figure 2A, the elements of Bi, Mo, and O exist in Bi_2_MoO_6_ samples, whereas a new element of sulfur appeared after the reaction between Bi_2_MoO_6_ and L-Cys. Peaks in Bi 4f spectra in Figure 2B showed that two main peaks at 159.0 and 164.3 eV belong to Bi 4f_5/2_ and Bi 4f_7/2_ in Bi_2_MoO_6_ (Jia et al., 2018), shifted to 159.3 and 164.6 eV after the chemical reaction. This chemical shift originated from the formation of new bonds between bismuth and sulfur which changed the original chemical environment of bismuth atoms. The high-resolution XPS spectra of Mo 3d, S 2p, and O 1s of Bi_2_MoO_6_ after reacting with L-Cys were also conducted. The binding energy at 232.3, 235.4, 159.2, 164.4, and 531.1 eV pictured in Figures 2C–E were ascribed to Mo 3d_5/2_, Mo 3d_3/2_, S 2p_3/2_, S 2p_1/2_, and O 1 s, respectively. The result further witnessed the *in situ* formation of Bi_2_S_3_ on Bi_2_MoO_6_ (Li et al., 2020).

**FIGURE 3:**
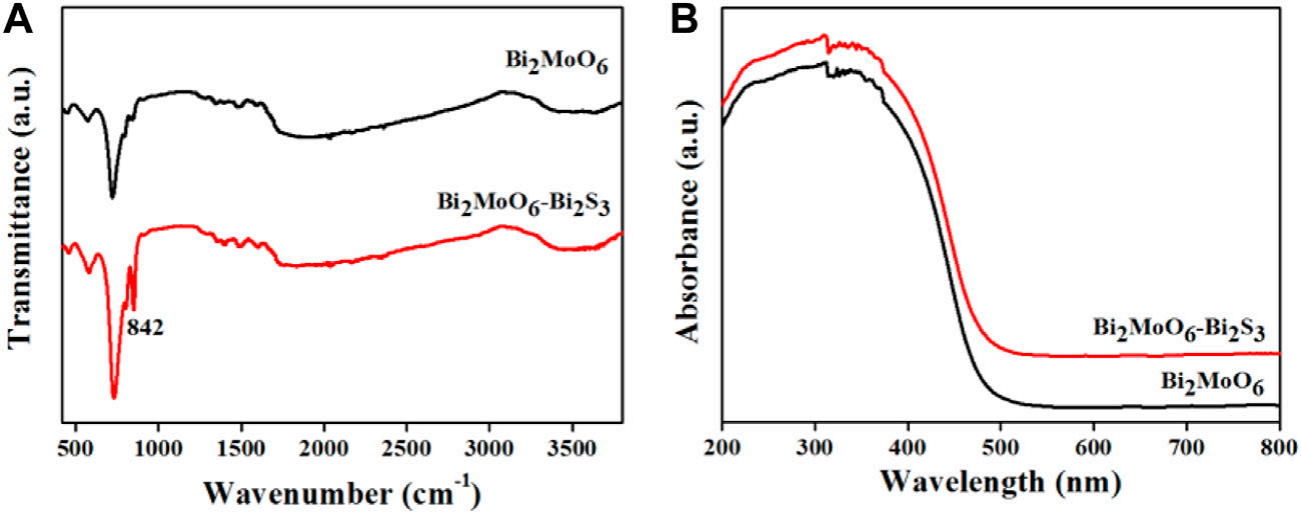
FT-IR spectra of Bi_2_MoO_6_ and Bi_2_MoO_6_–Bi_2_S_3_ **(A)**; UV-vis DRS of Bi_2_MoO_6_ and Bi_2_MoO_6_–Bi_2_S_3_ **(B)**.

The optical property of Bi_2_MoO_6_ before and after reacting with L-Cys was studied by FT-IR spectroscopy and UV-vis DRS. As can be seen from Figure 3A, the characteristic peak at 712 cm^−1^ existed both in the FT-IR spectrum of Bi_2_MoO_6_ and that after reacting with L-Cys, attributing to the symmetrical tensile vibration of the top oxygen atom of MoO_6_^6−^ (Zhang et al., 2010; Li et al., 2014a; Tian et al., 2015). Compared with the FT-IR spectrum of Bi_2_MoO_6_, a new peak at 842 cm^−1^ appeared in the chart of Bi_2_MoO_6_ after the reaction with L-Cys. This new peak corresponds to the stretching vibration of Bi–S, indicative of the formation of Bi_2_S_3_ through the reaction between Bi_2_MoO_6_ and L-Cys (Zhao et al., 2017). The UV-vis DRS in Figure 3B suggested that the formation of Bi_2_MoO_6_–Bi_2_S_3_ widened the absorption range of the light irradiation and thus is benefit for the subsequent PEC analysis.

### Condition Optimizations

As a photoactive material to construct the photoelectrode, the concentration of Bi_2_MoO_6_ plays a crucial effect on the PEC performance of the sensor. The photocurrent signal of the Bi_2_MoO_6_/ITO electrode constructed with varied concentration of Bi_2_MoO_6_ was recorded, and the photocurrent response reached a maximum value when the concentration of Bi_2_MoO_6_ was 3 mg ml^−1^ (Supplementary Figure S2). So, 3 mg ml^−1^ Bi_2_MoO_6_ was used for the subsequent experiments. In addition, the reaction time of Bi_2_MoO_6_ with L-Cys was optimized. According to Supplementary Figure S3, the photocurrent response gradually enhanced with the increase of reaction time, but the signal tended to stabilize when the reaction time reached 30 min. Therefore, 30 min was used as the reaction time.

### Electrochemical and Photoelectrochemical Characterizations

**FIGURE 4:**
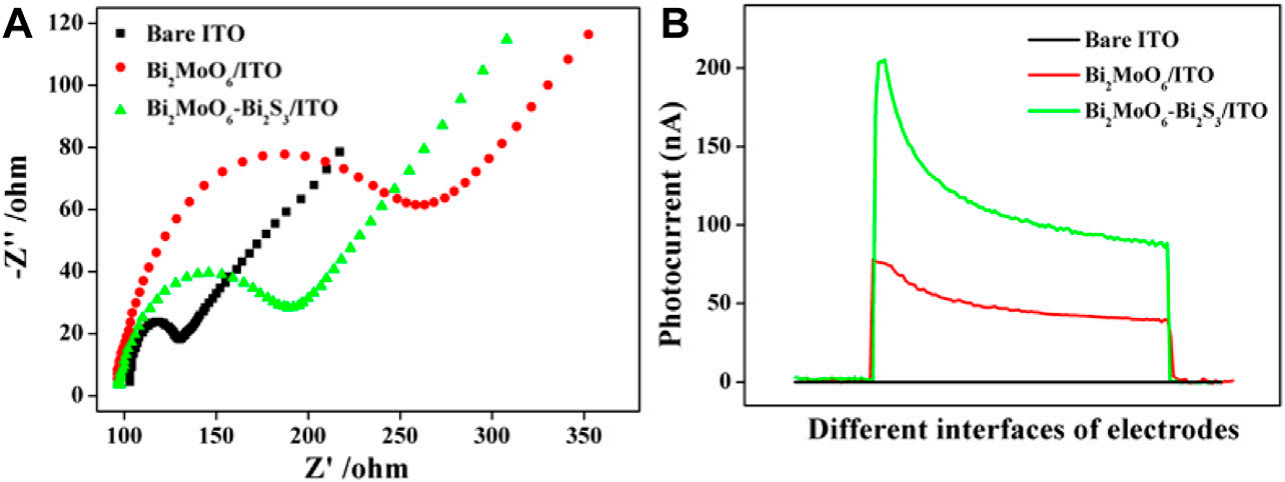
EIS **(A)** and photocurrent intensity **(B)** of bare ITO, Bi_2_MoO_6_/ITO, and Bi_2_MoO_6_/ITO after reacting with L-Cys.

**SCHEME 2:**
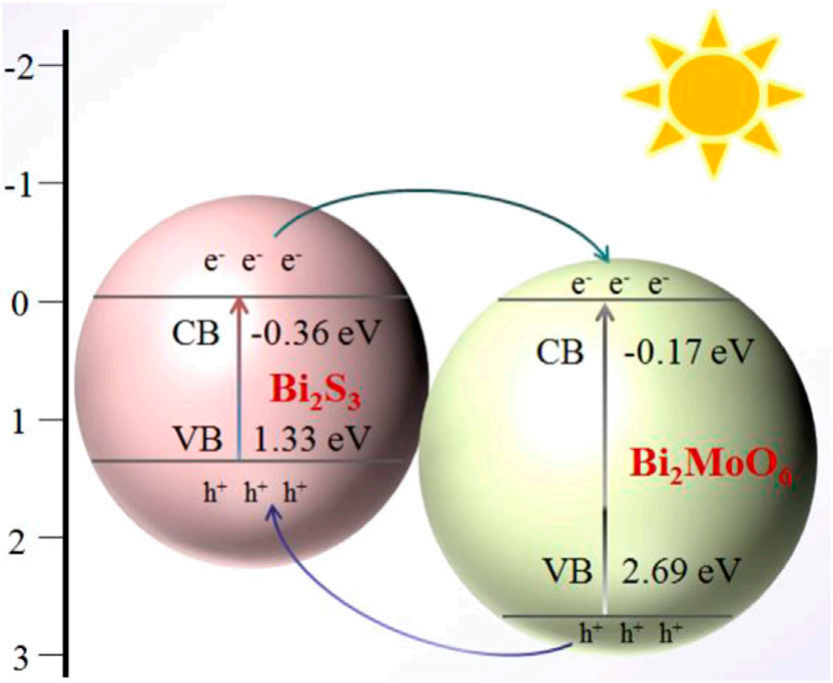
Charge transfer of Bi_2_MoO_6_–Bi_2_S_3_ under visible light irradiation.

To explore the interfacial electrochemical behavior of the biosensor, EIS analysis was conducted. As seen from Figure 4A, the bared ITO electrode displayed a small electron-transfer resistance (*R*_et_), whereas the Bi_2_MoO_6_/ITO electrode gave an increased *R*_et_ because the coating of the semiconductor impedes the electron transfer. After Bi_2_MoO_6_/ITO was incubated with L-Cys, the *R*_et_ declined. This result may be because the *in situ* formation of Bi_2_S_3_ on the interface of Bi_2_MoO_6_/ITO improved the electrical conductivity of the electrode. The photocurrent responses of the sensor at different modification stages were also investigated. As illustrated in Figure 4B, almost no PEC response was shown on the bare ITO electrode, while an evident photocurrent response was observed when Bi_2_MoO_6_ was immobilized on the electrode. After reacting with L-Cys (10 μmol L^−1^), the Bi_2_MoO_6_/ITO electrode gave a much stronger photocurrent response. This is because the compact heterostructure formed between Bi_2_S_3_ and Bi_2_MoO_6_ by *in situ* formation of Bi_2_S_3_ on Bi_2_MoO_6_ and the matchable band-edge levels of Bi_2_MoO_6_ and Bi_2_S_3_ could effectively accelerate the transfer of the photo-excited charge carriers. The valence band (VB) and conduction band (CB) energy levels of Bi_2_MoO_6_ and Bi_2_S_3_ were determined by the electrochemical method (Supplementary Figure S4), and the charge transfer in Bi_2_MoO_6_–Bi_2_S_3_ heterostructure is illustrated in Scheme 2. Under the light irradiation, the photo-generated electrons in the CB of Bi_2_S_3_ (−0.36 eV) easily transferred to the CB of Bi_2_MoO_6_ (−0.17 eV), whereas the holes in the VB of Bi_2_MoO_6_ (2.69 eV) moved to the VB of Bi_2_S_3_ (1.33 eV).

### Analytical Performance

**FIGURE 5:**
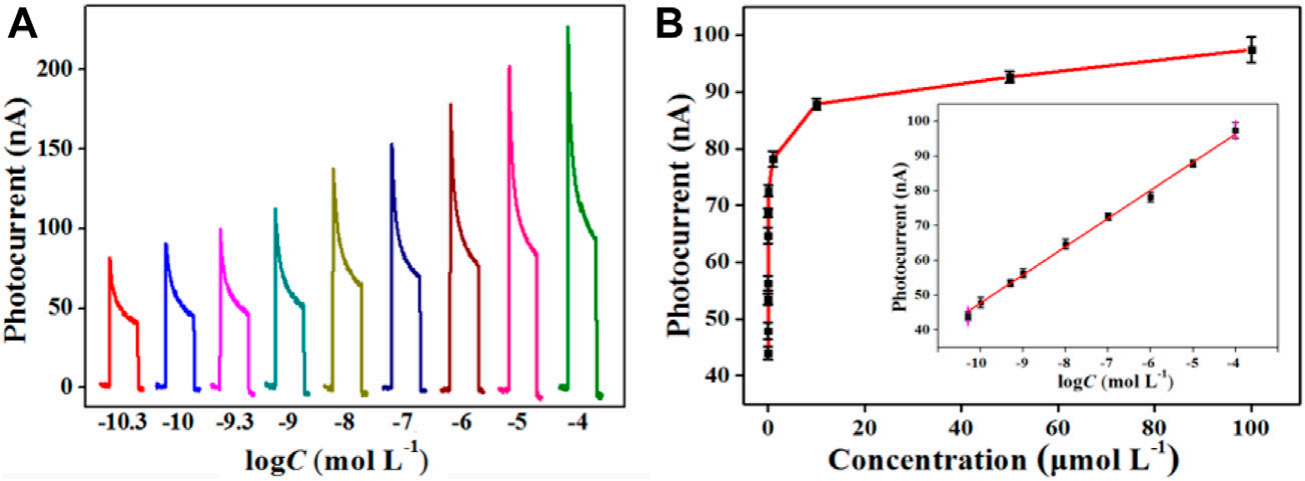
Photocurrent responses of Bi_2_MoO_6_/ITO corresponding to L-Cys with varied concentrations **(A)**; relationship between photocurrent changes and L-Cys concentrations **(B)**; insert of part B, calibration curve between photocurrents and the logarithm of the L-Cys concentrations.

The PEC response of the Bi_2_MoO_6_/ITO electrode toward L-Cys was explored. As depicted in Figure 5A, the photocurrent intensity enhanced along with the increase in L-Cys concentration. The reason of this variation trend may be that more L-Cys increased the amount of Bi_2_S_3_ *in situ* formed on the Bi_2_MoO_6_/ITO electrode, thus facilitating the charge transfer and boosting the photocurrent enhancement. As demonstrated in Figure 5B, the photocurrent intensity of the sensor showed a linear relationship with the logarithm of L-Cys concentrations when the concentrations varied in the range of 5.0 × 10^−11^–1.0 × 10^−4^ mol L^−1^. The linear equation is *I* = 128.7 + 8.1 log *C*_L-Cys_ (*R* ^2^ = 0.997). The limit of detection is 5.0 × 10^−12^ mol L^−1^. Compared with some reported methods, this method demonstrates high detection sensitivity and a wide linear range for L-Cys (Table 1). The excellent performance of the sensor can be attributed to the *in situ* formation of Bi_2_MoO_6_–Bi_2_S_3_ heterostructure, which possesses an excellent photoelectric response under light irradiation.

**TABLE 1:** Comparison between this method and the reported methods for L-Cys detection.

Method	Material	Linear range (mol L^−1^)	LOD (mol L^−1^)	Reference
Amperometry	Y_2_O_3_NPs/N-rGO	1.3 × 10^−6^–7.2 × 10^−4^	8.0 × 10^−7^	Yang et al. (2016)
Fluorescence	Carbon dots	0.0–3.0 × 10^−5^	3.4 × 10^−10^	Zong et al. (2014)
Colorimetry	CuNPs	0.0–2.5 × 10^−5^	1.0 × 10^−7^	Ahmed et al. (2016)
Electrochemiluminescence	PtNPs–RubRMs	1.0 × 10^−9^–5.0 × 10^−4^	3.3 × 10^−10^	Wu et al. (2019)
Ratiometric absorption	AuNPs–CS/PLNPs-IBA	1.0 × 10^−8^–5.5 × 10^−6^	2.2 × 10^−9^	Li et al. (2018)
Chronoamperometry	PB–AuNPs–Pd	3.0 × 10^−7^–4.0 × 10^−4^	1.8 × 10^−7^	Pandey et al. (2012)
Cyclic voltammetry	PPy/GQDs@PB	2.0 × 10^−7^–1.0 × 10^−3^	1.50 × 10^−7^	Wang et al. (2016)
PEC	Cu_2_SnS_3_@SnS_2_	1.0 × 10^−10^–3.0 × 10^−4^	6.8 × 10^−11^	Wang et al. (2020)
		1.0 × 10^−8^–1.0 × 10^−4^	8.5 × 10^−9^	
PEC	Bi_2_MoO_6_	5.0 × 10^−11^–1.0 × 10^−4^	5.0 × 10^−12^	This work

### Selectivity, Reproducibility, and Stability

**FIGURE 6:**
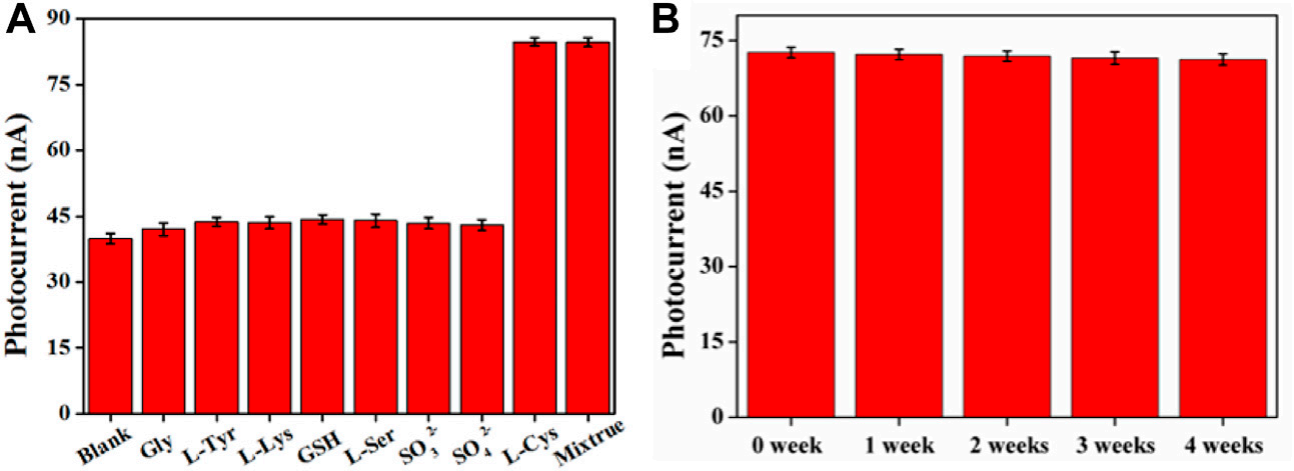
Selectivity **(A)** and stability **(B)** of the PEC sensor.

The selectivity of the sensor was evaluated by testing the PEC response of Bi_2_MoO_6_/ITO toward Gly, L-Tyr, L-Lys, GSH, L-Ser, SO_3_^2-^, and SO_4_^2-^ and the mixture of the aforementioned substances with L-Cys (all the aforementioned solutions have a concentration of 5 μmol L^−1^). As pictured in Figure 6A, the PEC responses of Bi_2_MoO_6_/ITO to Gly, L-Tyr, L-Lys, GSH, and L-Ser showed no obvious change compared with the blank solution, whereas the response of L-Cys as well as the mixture of the aforementioned interferents with L-Cys exhibited an obvious enhancement, thus demonstrating good selectivity. The reproducibility of the sensor was studied by intra-assay and inter-assay of 10 μmol L^−1^ L-Cys. The relative standard deviations (RSDs) of intra-assay by using five Bi_2_MoO_6_/ITO electrodes in the same batch and inter-assay of the electrodes in different batches were 3.0 and 4.2%, respectively, indicating good reproducibility of the sensor. In addition, the photocurrent response of Bi_2_MoO_6_/ITO for 100 nmol L^−1^ L-Cys within 4 weeks of storage was investigated to study the stability of the sensor. As shown in Figure 6B, the photocurrents show negligible change with RSDs less than 5.1%. The signal of this system for 15 cycles was monitored. In Supplementary Figure S5, the photocurrent was stable with a RSD of 3.2%. The data indicate the good stability of the sensor.

### Applications

To explore the practical application of the sensor, seven undiluted human serum samples from Xinyang Central Hospital were measured. As listed in Supplementary Table S1, compared with the reference method (enzymatic cycling) used by the hospital, the relative errors between the reference method and this method are less than 6.1%, and the RSDs are no more than 6.2%. In addition, the standard addition test results suggest that the recoveries of L-Cys are in the range of 90.0–110.0% with RSDs less than 6.8%, as shown in Supplementary Table S2. The aforementioned results show that this method has good accuracy and feasibility.

## Conclusion

In summary, a facile and signal-increased PEC sensor for L-Cys detection was developed based on the *in situ* formation of Bi_2_MoO_6_–Bi_2_S_3_ heterostructure. In virtue of the chemical reaction between L-Cys and Bi_2_MoO_6_, Bi_2_S_3_ was formed *in situ* on the surface of Bi_2_MoO_6_, and the signal-increased sensing system endowed the sensor with high sensitivity. The Bi_2_MoO_6_–Bi_2_S_3_ heterostructure showed effective photoelectric conversion efficiency and thus demonstrated sensitive photocurrent response under light irradiation. Thanks to the fine performance of the Bi_2_MoO_6_–Bi_2_S_3_ heterostructure, the sensor for L-Cys achieved excellent performance in sensitivity, selectivity, and stability. The proposed method based on the *in situ* growth reaction not only proposes a new strategy for L-Cys detection but also opens up a new perspective for PEC bioanalysis.

## Data Availability

The original contributions presented in the study are included in the article/Supplementary Material; further inquiries can be directed to the corresponding authors.
