# Supplementary material for: In Situ Formation of Bi2MoO6-Bi2S3 Heterostructure: A Proof-Of-Concept Study for Photoelectrochemical Bioassay of l-Cysteine
Source: Front Chem. 2022 May 18;10:845617. doi: 10.3389/fchem.2022.845617 (PMC9158332; doi:10.3389/fchem.2022.845617)
Supplement: Supplementary file 1 [file DataSheet1.docx]

Supplementary Material

In situ formation of Bi_2_MoO_6_-Bi_2_S_3_ heterostructure: A proof-of-concept study for photoelectrochemical bioassay of L-cysteine

**Hui-Jin Xiao^1^, Xiao-Jing Liao^1^, Hui Wang****^1^, Shu-Wei Ren^2^, Jun-Tao Cao^1*^, and Yan-Ming Liu^1*^**

^1^College of Chemistry and Chemical Engineering, Xinyang Key Laboratory of Functional Nanomaterials for Bioanalysis, Xinyang Normal University, Xinyang, China

^2^Xinyang Central Hospital, Xinyang, China

*** Correspondence:**

*Email: [liuym9518@sina.com](mailto:liuym9518@sina.com)

*Email: jtcao11@163.com


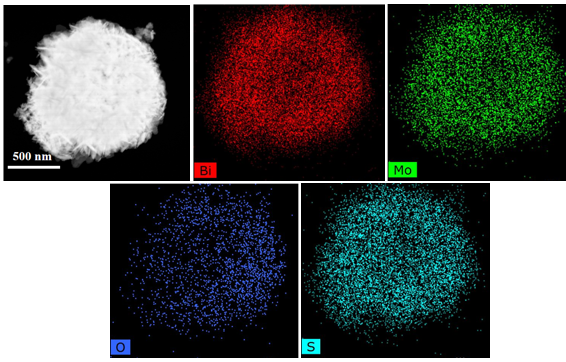


**Fig. S1.** Elemental mapping images of Bi_2_MoO_6_ after reacting with L-Cys.


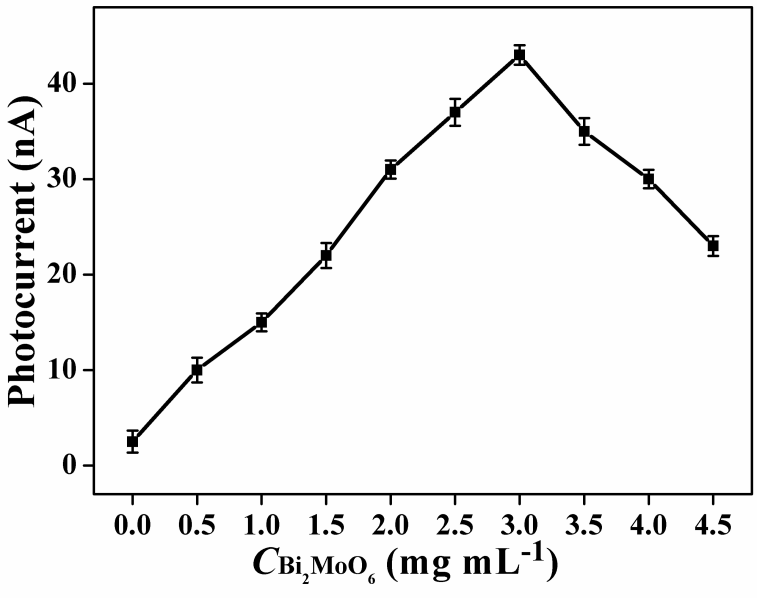


**Fig. S2.** Photocurrent responses of Bi_2_MoO_6_ with different concentrations in PBS solution (0.01 M, pH 7.4) containing 0.1 M AA.

**
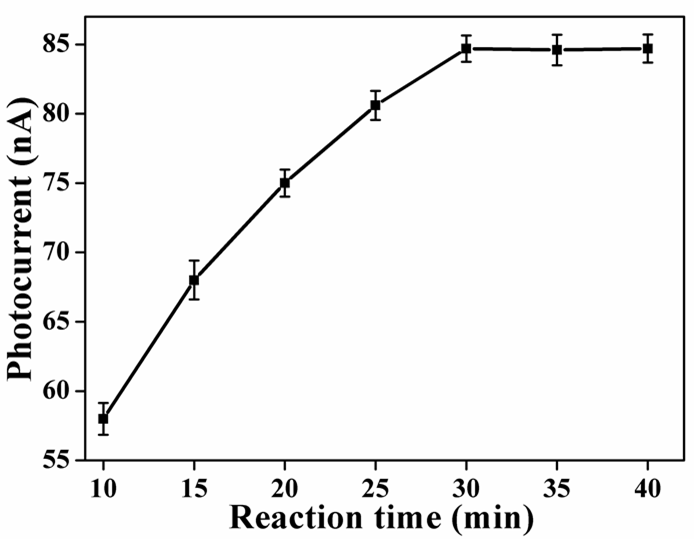
**

**Fig. S3.** Photocurrent responses of 3 mg mL^-1^Bi_2_MoO_6_ reacting with 5 µM L-Cys under different reaction time.


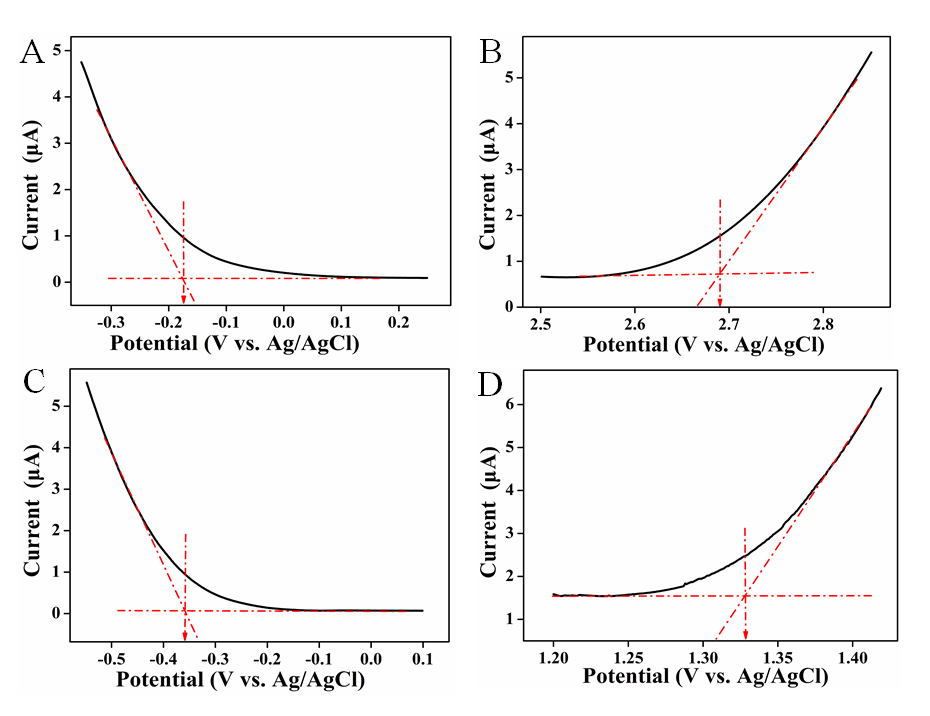


**Fig. S4.** Linear sweep voltammetry to determine the conduction bands (CB) and valence bands (VB) of Bi_2_MoO_6_ and Bi_2_S_3_ at a scan rate of 5 mV s^-1^ in 0.01 M PBS (pH = 7.4).

Excess L-Cys was incubated with Bi_2_MoO_6_/ITO to generate Bi_2_S_3_/ITO in situ. In addition, the conduction bands and valence bands of Bi_2_MoO_6_ and Bi_2_S3 were determined by linear sweep voltammetry (LSV). As presented in Fig. S3, the conduction band minimum (CBM) value and valence band maximum (VBM) value of Bi_2_MoO_6_ are -0.17 eV and 2.69 eV (vs. Ag/AgCl) (Fig. S3A and Fig. S3B), the CBM value and VBM value of Bi_2_S_3_ are -0.36 eV and 1.33 eV (vs. Ag/AgCl) (Fig. S3C and Fig. S3D). Consequently, the Eg (E_CB_ = E_VB_ - Eg) values of Bi_2_MoO_6_ and Bi_2_S_3_ were calculated to be 2.86 eV and 1.69 eV, respectively.


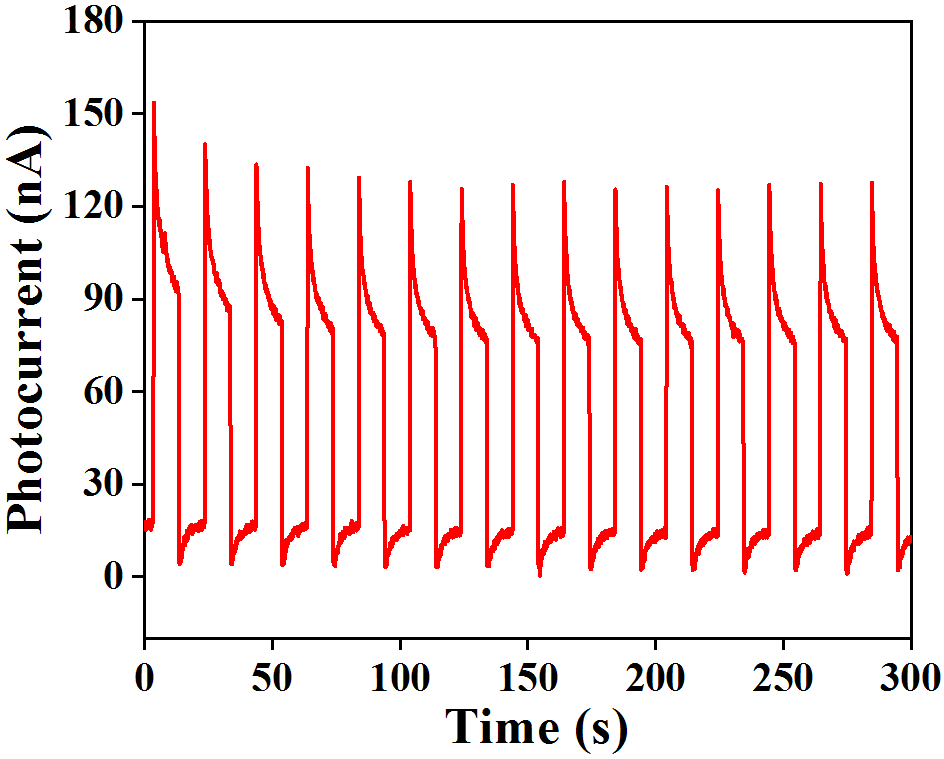


**Fig. S5.** Photocurrent responses of Bi_2_MoO_6_/ITO corresponding to 0.1 μM L-Cys.

**Table S1.** Analytical results for L-Cys in human serum samples

| Serum  samples No. | Reference method  (μmol L^-1^) | This work  (μmol L^-1^) | Relative errors  (%) | RSDs  (%, *n* = 3) |
| --- | --- | --- | --- | --- |
| 1 | 13.1 | 13.8 | 5.3 | 3.2 |
| 2 | 43.7 | 41.7 | -4.6 | 4.5 |
| 3 | 10.1 | 10.6 | 5.0 | 6.2 |
| 4 | 5.4 | 5.1 | -5.6 | 4.3 |
| 5 | 6.6 | 7.0 | 6.1 | 5.2 |
| 6 | 23.1 | 23.9 | 3.5 | 3.6 |
| 7 | 8.5 | 8.0 | -5.9 | 4.7 |

**Table S2.** Recoveries of L-Cys in two human serum samples

| Serum  samples No. | Found  (μmol L^-1^) | Added  (μmol L^-1^) | Total found  (μmol L^-1^) | Recovery  (%) | RSDs  (%, *n* = 3) |
| --- | --- | --- | --- | --- | --- |
| 1 | 5.4 | 0.10 | 5.49 | 90.0 | 5.4 |
|  |  | 5.00 | 10.32 | 98.4 | 6.2 |
|  |  | 25.0 | 29.85 | 97.8 | 4.6 |
| 2 | 23.1 | 0.10 | 23.21 | 110.0 | 5.7 |
|  |  | 5.00 | 27.90 | 96.0 | 6.8 |
|  |  | 25.0 | 48.60 | 102.0 | 3.9 |
